# Congenital Heart Disease: A Growing Population with Challenges to Patients and Providers

**DOI:** 10.3390/jpm13101442

**Published:** 2023-09-28

**Authors:** David S. Majdalany

**Affiliations:** Department of Cardiovascular Medicine, Mayo Clinic, 13400 East Shea Boulevard, Scottsdale, AZ 85259, USA; majdalany.david@mayo.edu

Congenital heart disease (CHD) is the most frequent disorder encountered in the newborn period and the most frequent cause of infant mortality [1]. It comprises 0.8–1.2% of all live births and more than 85% of the patients survive into adulthood thanks to the advances in diagnostic testing, pediatric cardiac care, timely surgical interventions, and catheter-based interventions which have been refined over time [2,3]. In fact, there are more adults with CHD than children with estimates of nearly 50 million adults with CHD worldwide with an expected steady increase [4,5].

In the United States, caring for patients with CHD currently requires 2 additional years of subspecialized training after completing a Cardiology fellowship as there is a broad range of cardiac anomalies with each exhibiting anatomic heterogeneity, unique natural history, amenability to surgical or percutaneous interventions, and variable long-term sequelae. In addition to specialized training in the field of CHD, caring for these ever-growing complex patients requires access to tertiary care centers with multidisciplinary teams with expertise in CHD surgery, interventional cardiology, pediatric cardiology, intensive care, cardiac anesthesia, cardiac imaging, electrophysiology, heart failure and transplant, pulmonary hypertension, genetics, cardio-obstetrics, maternal fetal medicine, and psychiatry. The CHD population typically requires lifelong care as many of the interventions are palliative and the patients may require re-intervention in the future. With the care being very resource-intense, the health system will need to be accepting of the financial burden optimal care for this underserved segment of the population will necessitate. 

Despite the amazing breakthroughs in medical diagnosis and intervention, there are still challenges encountered and room for improvement in the care of patients with CHD. The challenges are not only for the patients with CHD but also for the cardiac surgeons, the general cardiologists, and the CHD cardiologists [6].

The challenges for CHD patients stem from the false sense of security that the interventions they underwent were curative and no further cardiac follow-up is warranted. This may be due an unawareness and possible sub-optimal education that the interventions in most cases are palliative and may be a “temporary fixes”, especially in moderate to high complexity forms of CHD such as tetralogy of Fallot and transposition of the great arteries. Common long-term sequelae of CHD intervention include heart failure, arrhythmias, reduced functional capacity, valvular disease, or dysfunction of conduits, baffles, and prosthetic valves. For example, patients with tetralogy of Fallot would typically undergo surgical intervention which includes patch closure of the ventricular septal defect and relief of the right ventricular obstruction. The latter may include valvotomy/valvectomy of the pulmonary valve and patch enlargement of the right ventricular outflow tract or pulmonary artery. This intervention would result in relief of the cyanosis and permit the child to grow to adulthood albeit in the setting of important pulmonary valve regurgitation which initially may not cause any symptoms or be routinely detected on clinical exam. However, as the patient reaches adulthood, unrecognized significant pulmonary valve regurgitation may lead to progressive right ventricular enlargement which may be irreversible, right-sided heart failure, and dysrhythmias. Moreover, another challenge patients would encounter is suboptimal access to tertiary care centers with multidisciplinary expertise in CHD. Even when they are able to arrange follow-up, lack of medical coverage or being under-insured is another hurdle patients would encounter as their evaluations which may include serial cardiac imaging, cardiac catheterization, and seeing multiple subspecialists which can be quite expensive and discouragingly beyond their budget. 

Despite tremendous surgical advancements for patients with CHD, challenges still exist for surgeons. With the heterogeneous nature of CHD and a continuum of complexities, the CHD patients are best taken care of by surgeons with additional training in congenital heart surgery. Unfortunately, there is a paucity of experienced CHD surgeons. Moreover, since the surgical interventions tend to be palliative except for the simple ligation of a patent ductus arteriosus, surgeons are faced with the challenges of multiple reoperations: redo sternotomies would require increased operative time with clearing of extensive scar tissues; there are risks of arterial or cardiac injury as the great vessels may adhere to the sternum; and vascular access for cardiopulmonary bypass may be limited. With the aging population with an increased prevalence of acquired heart disease, metabolic syndrome, and increased co-morbidities including renal and hepatic disease, the surgeons have to take into account the resultant elevated peri-operative risks when deciding to offer the patient further surgical interventions [6]. Finally, a key to surgical success is the need for excellent intensive post-operative care made up of intensivists and nursing staff who are familiar with and comfortable in managing postoperative CHD patients with their inherent unique cardiac anatomy, physiologies, and hemodynamics. 

Challenges do exist for both the general cardiologist as well as the CHD cardiologist. The general cardiologist may have had limited exposure to CHD patients, the confusing terminology of the diagnoses and surgical interventions, and the long-term sequelae of their palliated disease. They may miss a radial–femoral delay or discordant upper and lower extremity blood pressure readings in a young hypertensive patient with coarctation as this may not be part of the routine exam in a busy community practice. Patients with cyanotic congenital heart disease are expected to have secondary erythrocytosis which is compensatory; elevated hemoglobin levels may concern the primary providers and prompt inappropriate phlebotomies which are harmful to CHD patients and predispose them to iron deficiency anemia and increased risk of stroke. Imaging patients with CHD post multiple surgical interventions may be challenging to the general cardiologists as acoustic windows may be limited on echocardiography and they may require supplementary tomographic cardiac imaging and CHD expertise for optimal interpretation of the findings. Finally, as mentioned earlier, general cardiologists may be under the misconception that the surgical interventions in CHD patients are “totally corrective” and may fail to refer patients for appropriate surveillance in a timely fashion [6]; with late referrals the only option for the patients may have would be consideration of advanced heart failure therapies or heart transplant.

Obstacles do exist for the CHD-trained cardiologists. The challenges commence as soon as they complete their general cardiology fellowships as few programs offer combined five-year programs that merge Pediatric and Adult Cardiology. Otherwise, the interested candidates have to apply and match for limited CHD fellowship spots and dedicate two additional years to acquire the necessary training to be eligible for board certification. Consequently, the supply of such cardiologists is not meeting the rising demand for their skills with the growing and aging CHD population. In addition, finding employment post-training is a big hurdle as consultant positions are limited and many medical centers may lack the infrastructure and resources needed to build a successful CHD program. Building a CHD program requires successful pediatric and adult cardiology medical and surgical teams and offers the patients the multidisciplinary care they would need such as electrophysiology, interventional cardiology, pulmonary hypertension, heart failure, intensive care, mental health, etc. The CHD patients should be transferred to the appropriate adult providers to meet the changing spectrum of their cardiac disease as they may demonstrate features of acquired heart disease such as coronary artery disease. 

Even when the CHD cardiologists are fortunate to secure a consultant position, they have to face a balance between “quality” of care vs. “quantity” of care given pressures for revenue productivity as they are pushed to see more and more patients in less time slots which can compromise the care and result in patient dissatisfaction. The hiring medical institution needs to be committed to providing administrative and financial support as well as different revenue target goals and longer time for patient visits for the CHD providers. Collecting the prior medical records for CHD patients is an obstacle as some may no longer be available depending on the era of the intervention or the records are unobtainable transforming the CHD cardiologist into a medical detective. The visits for CHD patients are time-intensive with time spent reviewing piles of prior medical records, time educating patients about the long-term sequelae of their cardiac disease and need for follow-up, thorough documentation of the extensive visit data, coordinating the care amongst the different needed subspecialities, responding to the patient’s post-visit questions, corresponding with insurance companies to approve coverage for the required testing that tend to be costly, and liaising with the patient’s employment regarding work restrictions and potential disability. The CHD cardiologist needs to fulfill his role as an educator not only for the patients but also for the medical trainees, nurses, sonographers, and other colleagues. As there is a paucity of funding in research, academic CHD cardiologists would need to dedicate time to organize a database with the support of their medical institution and partake in multicenter projects to advance the field further and expand the CHD knowledge base. 

Patients with congenital heart disease are growing in numbers and living longer due to improvements in diagnostic testing and timely interventions. Challenges exist for this underserved population as well as for their medical and surgical providers. Optimizing their care depends on improving patient and provider education and awareness, institutional support for and investment in the limited numbers of medical providers, and multicenter prospective studies to guide the evolving medical and interventional aspects of the care of this complex and heterogeneous cohort of patients. 
